# Nanowire melting modes during the solid–liquid phase transition: theory and molecular dynamics simulations

**DOI:** 10.1038/s41598-022-24654-z

**Published:** 2022-11-21

**Authors:** Kannan M. Ridings, Shaun C. Hendy

**Affiliations:** 1 The MacDiarmid Institute for Advanced Materials and Nanotechnology, Department of Physics, The University of Auckland, Auckland, 1010 New Zealand; 2 Toha Foundry, Auckland, 1025 New Zealand

**Keywords:** Nanoscale materials, Theory and computation, Nanoscale devices, Nanoscale materials

## Abstract

Molecular dynamics simulations have shown that after initial surface melting, nanowires can melt via two mechanisms: an interface front moves towards the wire centre; the growth of instabilities at the interface can cause the solid to pinch-off and breakup. By perturbing a capillary fluctuation model describing the interface kinetics, we show when each mechanism is preferred and compare the results to molecular dynamics simulation. A Plateau-Rayleigh-type of instability is found and suggests longer nanowires will melt via an instability mechanism, whereas in shorter nanowires the melting front will move closer to the centre before the solid pinch-off can initiate. Simulations support this theory; preferred modes that destabilise the interface are proportional to the wire length, with longer nanowires preferring to pinch-off and melt; shorter wires have a more stable interface close to their melting temperature, and prefer to melt via an interface front that moves towards the wire centre.

## Introduction

Nanostructured objects have lower stability with respect to their molten phase due to large surface area to volume ratios^1–3^. In the case of nanowires, their stability has been studied at elevated temperatures both experimentally^4–6^ and theoretically^7,8^ indicating the presence of Plateau-Rayleigh (PR) type of instabilities which can cause a nanowire to neck and breakup into a chain of nanospheres. In fact, PR like instabilities have been used as a means of self-assembly of chains of nanospheres for several different initial geometries ranging from rings^9^, wires^10^, and thin films^11,12^. PR theory generally predicts that the wavelength $\lambda$ of the perturbations which cause a liquid wire to become unstable are proportional to the initial wire circumference (i.e. wires become unstable when $\lambda _{\text {c}} > 2 \pi R_0$). Moreover, linear stability analysis predicts a preferred wavelength that will drive a liquid wire to breakup. Much work has been done in regards to nanocluster stability during solid-liquid coexistence^13^, and the stability of liquid nanojets^14,15^, nanocylinders^16^, and even in cylindrical metal alloys^17^, relatively few studies address the stability of the solid core of nanowires close to their melting point.

It was found that for finite-sized cylinders during phase coexistence, differences in curvature and fluctuations would lead to the formation of random breaches at the material interface, causing the growth of instabilities which lead to the melting of the solid^18^. For finite-sized boxes, different crystal geometries are realised by overcoming nucleation barriers, where a crystal nucleus surrounded by its own fluid could change from a slab geometry to a cylinder, and then to a solid droplet. It suggests that the solid prefers metastable forms as the box approaches the freezing (or melting) density^19^. This indicates that stable geometries of a given phase depend on the volume fraction of said phase (or medium) to the system volume^20–22^. Recent work has studied the breakage of gold nanofilaments connecting two nanoparticles where the filaments connecting the two nanoparticles would break apart by Joule heating^23^. Moreover, it was observed that the temperature at the breakage point had a strong dependence on the filament width, and had a dependence on the length in some, but not all cases^23^.

The thermally induced breaking of nanowires becomes important when considering the role they play in devices that utilise nanowire networks. Heat can be generated in nanowire networks via current passing through the network, and as such can influence the morphology and breakup of the nanowires making up the network^24,25^. This could be a hindrance to device stability, where it is important to understand the limitations of interconnecting materials like nanowires.

In this paper, we investigate the stability of metal nanowires as they approach their melting temperature for copper nanowires of varying lengths and radii. To describe the nanowire stability, we perturb a capillary fluctuation model that describes the kinetics of the solid-liquid interface. The model is then tested against molecular dynamics (MD) simulations, where it is found that longer nanowires are more unstable with respect to the melt.

## Results

### Capillary fluctuation model

**Figure 1:**
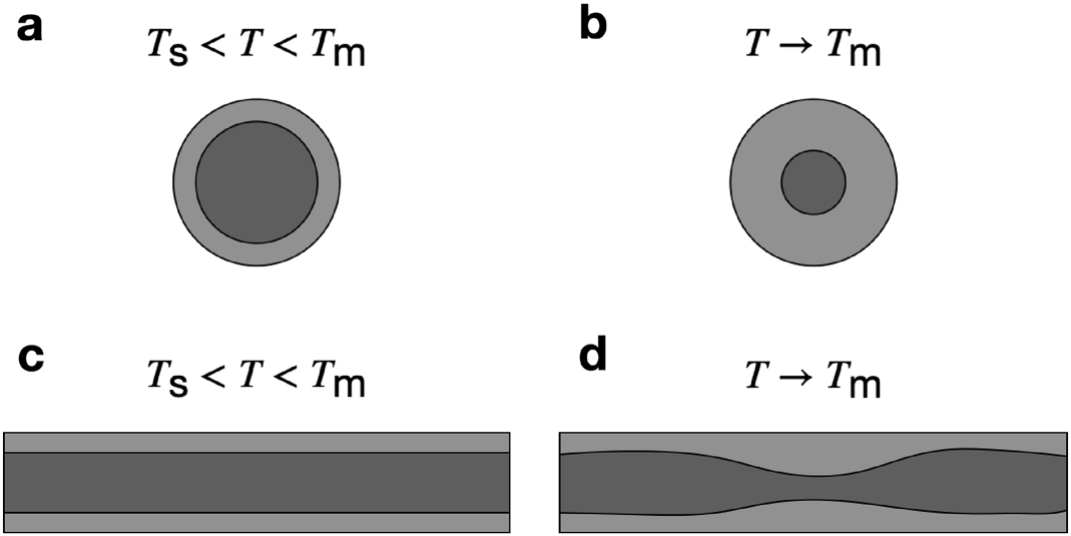
Two different melting modes of nanowires where light grey represents the liquid, while dark grey represents the solid. Panels (**a**)–(**b**) represent the moving interface front mechanism, whereas panels (**c**)–(**d**) show melting initiated via an instability at the solid-liquid interface.

Melting at the nanoscale is thought to initiate at the surface, and move from the outside inwards, with the interface consuming the solid as it melts. However, observations in nanowires show the solid will begin to neck and breakup as the nanowire approaches its melting point $T_{\text {m}}$^8^. Figure 1a shows a top-down view of a nanowire at a temperature *T* that sits between its surface melting temperature $T_{\text {s}}$ and bulk melting temperature $T_{\text {m}}$ (where $T_{\text {m}}$ is the bulk melting temperature of finite-sized nanowire). Figure 1b shows that as $T \rightarrow T_{\text {m}}$ the solid is consumed radially as the interface moves towards the wire centre. Figure. 1c, d shows the melting instability mechanism. Figure 1c shows a side-on view of a nanowire where $T_{\text {s}}<T<T_{\text {m}}$. However, as $T \rightarrow T_{\text {m}}$, rather than the interface moving towards the centre, a portion of the solid begins to thin out and neck, as seen in Fig. 1d, initiating the solid breakup and causing the remaining solid to be consumed.

We first consider the Gibbs free energy difference per unit length in an infinitely long cylinder surrounded by its own melt close to $T_{\text {m}}$ (see^18^ supplementary theories)

1 $$\begin{aligned} \Delta G = - \pi r^2 L_{\text {v}}(\Delta T/T_c)+ 2 \pi r \gamma _{\text {sl}}. \end{aligned}$$

The value of *r* that minimizes this equation represents the equilibrium solid radius for an infinitely long nanowire in the vicinity of $T_{\text {m}}$

2 $$\begin{aligned} r^* = \frac{\gamma _{\text {sl}}T_c}{ L_{\text {v}} \Delta T}. \end{aligned}$$

**Figure 2:**
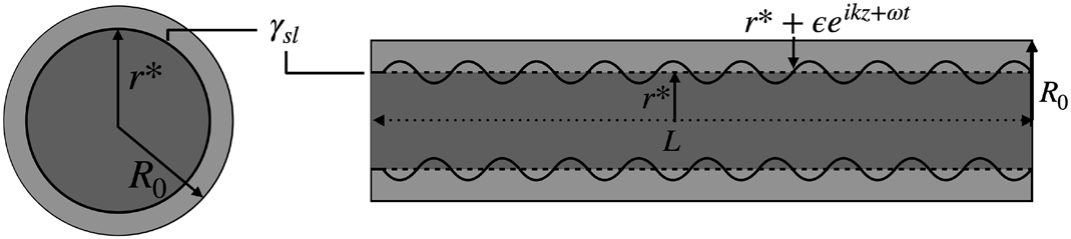
A surface melted nanowire of radius $R_0$ and length *L*. The solid core radius during solid-liquid phase coexistence is $r^*$. This surface has undergone a small perturbation in the form of a plane wave $r^* + \varepsilon e^{ikz + \omega t}$. $\gamma _{\text {sl}}$ represents the solid-liquid interfacial energy.

Here, $\gamma _{\text {sl}}$ represents the solid-liquid interfacial energy, $L_{\text {v}}$ is the bulk latent heat of melting per unit volume, $T_c$ melting temperature of the bulk materials and $\Delta T$ is the undercooling. Now we look at the interface velocity for a cylindrical nucleus^18^. For an infinite flat interface there is zero undercooling, and if $T<T_c$, the solid-liquid interface will propagate towards the liquid phase with a velocity *V*^26–29^

3 $$\begin{aligned} V = V_0\Big (1 - e^{-Q/k_b T}\Big ), \end{aligned}$$

where $V_0$ represents a maximum velocity that depends on temperature, *Q* is defined to be a thermodynamic driving force, and $k_b$ is Boltzmann’s constant. *Q* is defined as the difference between the solid and liquid phases per atom, so in a flat interface limit, it can be approximated as $Q \simeq L_{\text {v}} \Delta T / N\, T_c$, where *N* is the number density. Taking Eq. (3), substituting for *Q* and taking $T = T_c - \Delta T$, and expanding in the small undercooling limit the interface velocity can be linearised as

4 $$\begin{aligned} V = \frac{V_0 L_{\text {v}}}{N k_b T_c^2}\Delta T = \zeta \Delta T, \end{aligned}$$

where $\zeta$ is a kinetic coefficient. This gives the planar interface velocity in the small undercooling limit. We now consider the interface kinetics by looking at the dynamic behaviour of a cylindrical interface with a profile $\tilde{r}(z,t)$^18,30,31^

5 $$\begin{aligned} \frac{d\tilde{r}}{dt} = \zeta \Gamma \frac{d^2\tilde{r}}{dz^2} + \zeta \tilde{\eta } + \zeta \Delta T\Big (1 - \frac{r^*}{\tilde{r}}\Big ), \end{aligned}$$

where $\Gamma = (\gamma _{\text {sl}} + \gamma _{\text {sl}}'')T_c/L_{\text {v}}$, with $\gamma _{\text {sl}} + \gamma _{\text {sl}}''$ the interfacial stiffness of the solid-liquid interface^32^. Assuming an isotropic solid-liquid interface and small anisotropy, this can be approximated as $\Gamma \approx \gamma _{\text {sl}}T_c/L_{\text {v}}$^31^. $\tilde{\eta }$ is a thermal noise term, and takes into account fluctuations about equilibrium and satisfies $\langle \tilde{\eta }(x,t)\tilde{\eta }^*(x',t')\rangle = {\mathscr {C}}\delta (x - x')\delta (t-t')$, where ${\mathscr {C}}$ is a constant, and the delta functions suggest the noise is uncorrelated in space and time. We perturb the solid-liquid interface by a small parameter $\varepsilon$, and express it as the surface $\tilde{r}(z,t) = r^* + \varepsilon e^{ikz + \omega t}$, with *k* and $\omega$ being the wavenumber and instability growth rate respectively (see in Fig. 2). The term $(r^* + \varepsilon e^{i k z + \omega t})^{-1}$ can be approximated as

6 $$\begin{aligned} \frac{1}{r^* + \varepsilon e^{i k z + \omega t}} \simeq \frac{1}{r^*} - \frac{\varepsilon }{r^{*2}}e^{i k z + \omega t}. \end{aligned}$$

By substituting $\tilde{r}$ into Eq. (5), using the expression in Eq. (6), solving to ${\mathcal {O}}(\varepsilon )$, and using the definition of $r^*$ in Eq. (2) we recover

7 $$\begin{aligned} \omega = \frac{\zeta \Delta T}{r^*}\Big (1 - k^2 r^{*2} \Big ). \end{aligned}$$

A PR type instability can be found by observing that $\omega > 0$ when $kr^* < 1$, bringing us to the familiar solution

8 $$\begin{aligned} \lambda ^* > 2 \pi r^*. \end{aligned}$$

Combining Eqs. (8) and (2) and taking $\Delta T = T_c - T_{\text {m}}$, the interface will remain stable when $\omega < 0$ and leads to the moving interface front seen in Fig. 1a to b giving the condition

9 $$\begin{aligned} T_{\omega } < T_{\text {m}}, \end{aligned}$$

where we define $T_{\omega }=T_c\bigg (1 - \frac{2 \pi \gamma _{\text {sl}}}{ L_{\text {v}} \lambda ^*}\bigg )$. If $\omega > 0$ then $T_{\omega } > T_{\text {m}}$, and perturbations will grow to destabilise the solid-liquid interface, giving the scenario in Fig. 1c–d. If $\lambda ^* \propto L$ then $T_{\omega }$ becomes larger than $T_{\text {m}}$ quickly, giving a criteria describing when each melting mode is preferred.

Finally, we look at the equation for the bulk melting temperature of a cylindrically symmetric nanowire of radius $R_0$ from previous work^8^,

10 $$\begin{aligned} T_{\text {m}}=T_c\bigg (1 - \frac{4 \gamma _{\text {sl}}}{L_{\text {v}}R_0}\bigg (\frac{I_1(R_0/\xi )}{I_0(R_0/\xi ) + \kappa I_1(R_0/\xi )}\bigg )\bigg ), \end{aligned}$$

where $\kappa = \frac{1-\Delta \gamma /\gamma _{\text {sl}}}{1+\Delta \gamma /\gamma _{\text {sl}}}$ ($\Delta \gamma$ is the spreading parameter that determines the wettability of a material^8^), $\xi$ represents the correlation length, $I_0$ and $I_1$ represent modified Bessel functions of the first kind respectively. Equation (10) is found by solving a two-parabola Landau-type model for the free energy of a cylindrically symmetric nanowire^8^. Combining Eqs. (2) and (10), a equation for $r^*$ in terms of $R_0$ and interfacial energies $\kappa$ can be found

11 $$\begin{aligned} r^* = \frac{R_0}{4}\bigg ( \kappa + \frac{I_0(R_0/\xi )}{I_1(R_0/\xi )} \bigg ). \end{aligned}$$

## Methods

MD simulations were performed using LAMMPS^33^ using an embedded-atom-model (EAM) potential for copper^34^, with a bulk melting temperature of 1320 K. This potential was chosen because yielded good results for melting temperatures and dynamics^8^. Periodic boundary conditions in all directions were used, with the periodicity along the wire axis effectively simulating an infinitely long wire, and additionally suppress long-wavelength instabilities that may otherwise cause the wire to break apart prior to completely melting. As such, the maximum wavelength allowed by the system will be equal to the box size along the axis of the nanowire.

The equations of motion were integrated using a Verlet method using a timestep of 2.5 fs. The temperature was controlled with a Langevin thermostat, which effectively simulates Brownian motion, and is a appropriate choice of thermostat since To control the temperature a Langevin thermostat with a damping parameter of 1.0 $\hbox {ps}^{-1}$. This was to ensure a quick equilibration at each timestep of the simulation. The simulations were initialized at an initial temperature $T_i$ for 1.0 ns. Afterwards, a production phase for each wire was run from a temperature $T_i$ to a temperature $T_i + 1$. Then an equilibration phase around the temperature $T = T_i + 1$ was run. Each production phase was 0.40 ns, with an equilibration phase of 0.60 ns, creating an effective heating rate of around 1K/ns. This ensured us that at each temperature the wires were sufficiently close to equilibrium. See supplementary materials S2 and S3 for more information on computational details and methods used in this study.

### Molecular dynamics simulations

We begin with three nanowire radii $R_0$ of 22.0, 30.0, and 38.0 Å. We then select four lengths such that the aspect ratios $L/R_0$ of 5.0, 7.5, 15.0, and 25.0 were satisfied. These four wire lengths were chosen to ensure that $L_1 < L_{\text {crit}}$, $L_2 \simeq L_{\text {crit}}$, $L_3 > L_{\text {crit}}$ and $L_4 \gg L_{\text {crit}}$, where $L_{\text {crit}} \simeq 2 \pi R_0$, represents the wire length closest to the classical PR critical wavelength (perturbations grow when $\lambda _{\text {c}} > 2\pi R_0$). Each individual wire had 10–20 individual runs to gather statistics on the quantities of interest.

**Figure 3:**
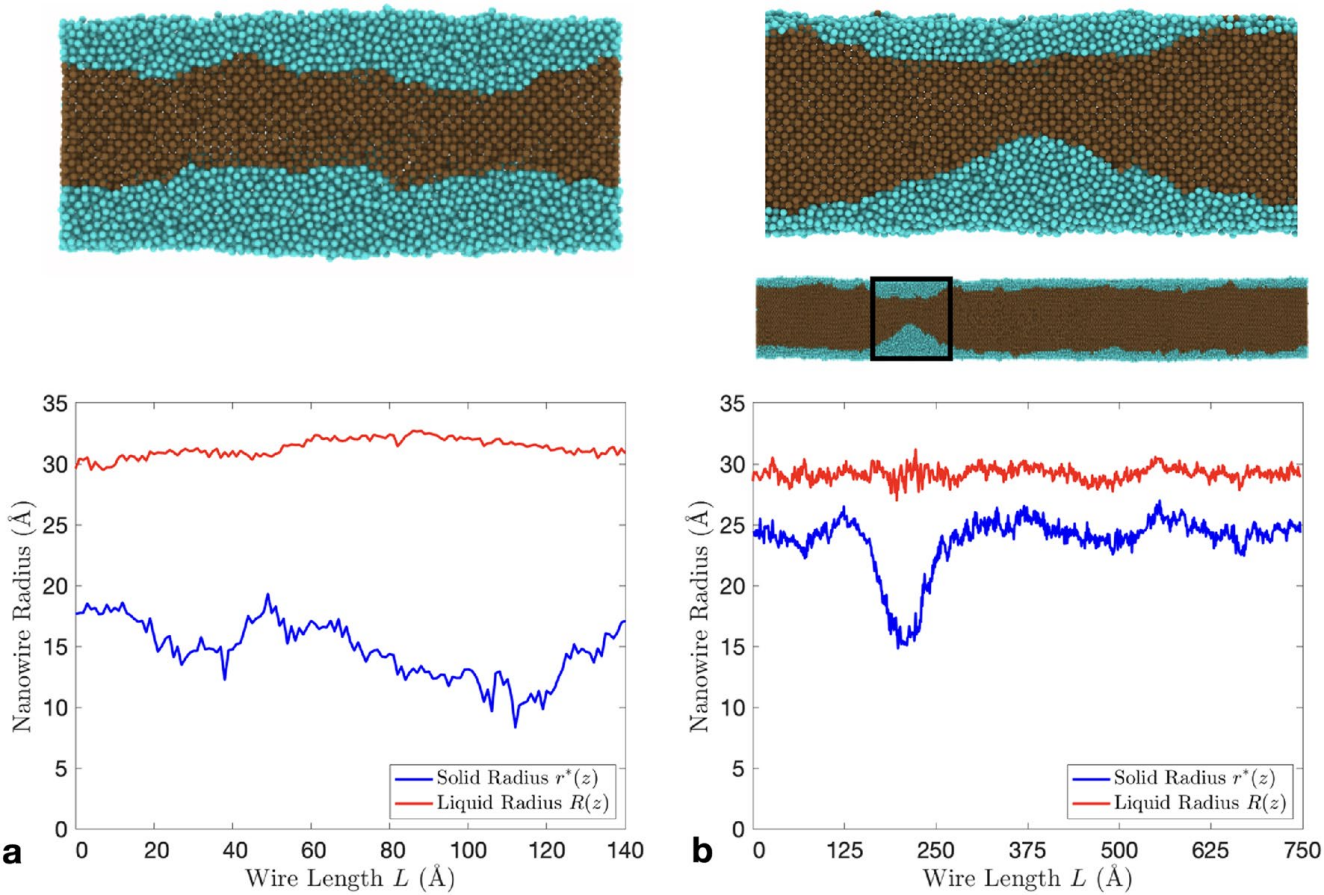
A plot showing the solid radius $r^*(z)$ for two surface melted nanowires both of radius $R_0 \approx 30$ Å and lengths 140 and 750 Å from (**a**)–(**b**) respectively. Solid blue lines represent the radius of the solid-liquid interface, while the solid red lines indicate the liquid interface. The solid atoms are coloured brown (darker) and the liquid atoms are coloured blue (lighter). The snapshots of the nanowires in (**a**) and (**b**) are both right before the solid core breaks apart. In (**a**) the size of the liquid nucleus is nearly half the nanowire radius, whereas in (**b**) the liquid nucleus only takes up a small portion of the total nanowire radius.

We can study the stability of the solid-liquid interface by examining when the solid core begins to pinch-off for two wires of the same radii, but different lengths, as shown in Fig. 3. As $T\rightarrow T_{\text {m}}$, the interface will either move towards the wire centre Fig. 3a or begin to pinch-off Fig. 3b. In Fig. 3a the size of the liquid nucleus is large compared to the much longer wire. The presence of solid atoms close to the liquid surface in Fig. 3b can be seen by the presence of a ‘noisy’ interface (red solid line). We will see in this section, wires with lengths that satisfy $L > 2\pi R_0$ will pinch-off and melt at a temperature consistently lower than wires with lengths $L \le 2\pi R_0$.

**Figure 4:**
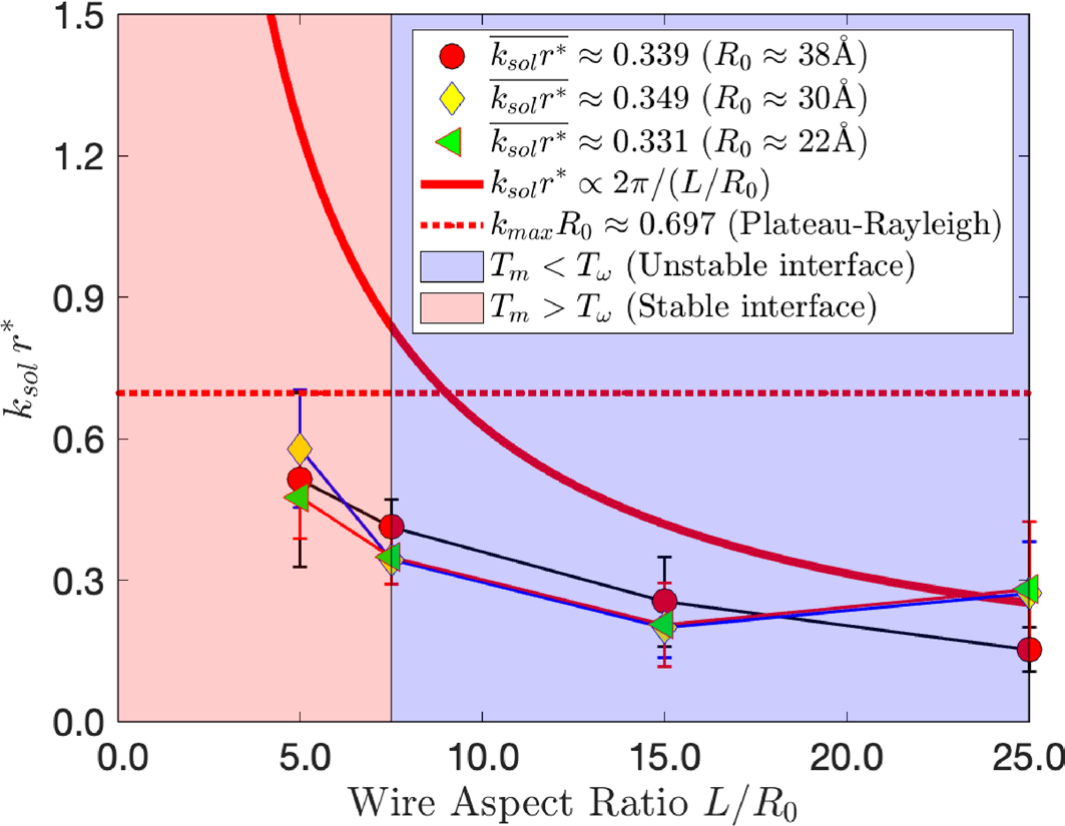
This figure shows how the wire aspect ratio affects the fastest growing modes. A thick red line plots $2 \pi /(L/R_0)$, which we assume is proportional to $k_{sol}r^*$. The light shaded area shows the region where the interface is stable at temperatures close to $T_{\text {m}}$ (radial mode), whereas the darker shaded region shows where the interface is expected to be unstable (instability mode).

To study the stability of the solid-liquid interface, the Fourier transform of the solid is taken to extract modes that destabilise the interface (see supplementary details S3). Figure 4 represents a stability diagram in terms of the fastest growing modes $k_{sol}r^*$ against wire aspect ratio $L/R_0$, where each $k_{sol}r^*$ is the averaged value of $kr^*$ that represents the maximum Fourier transform amplitude (see supplementary details Fig. S3). The modes $k_{sol}r^*$ for each wire aspect ratio are similar, which indicates destabilising modes depend strongly on the wire aspect ratio. As nanowires get shorter, modes that destabilise the interface approach unity, in violation of classical PR theory (see red dashed line). Also seen are two regimes that identify the preferred melting mode, as seen in Fig. 1. To the left (light pinkish region) we see the regime where $T_{\omega } < T_{\text {m}}$ which indicates the solid-liquid interface must move closer to the centre before the pinch-off can initiate. On the right (dark bluish region) we see the regime where $T_{\omega } > T_{\text {m}}$ and identify when the pinch-off melting mechanism is favoured. Observations from MD simulation agree with the theory developed, represented by Eqs. (7),  (8) and  (9). Longer wires will be more thermodynamically unstable since wire lengths will generally be greater than the circumference of the coexisting solid, as indicated by Eq. (8). Included in Fig. 4 is the scaling relation $k_{sol}r^* \propto \frac{2 \pi R_0}{L}$, which follows the trend observed in MD simulations, as well as predictions by classical PR theory which states $k_{max}R_0 \simeq 0.697$. For wires with $L<2\pi R_0$, $k_{sol}r^*$ approaches and exceeds unity, as predicted by the scaling relation, violating classical PR theory and indicating regions of interface stability. Moreover, we see that for higher wire aspect ratios, PR theory overpredicts the fastest growing modes in the solid. This has been previously reported when examining the stability of liquid nanojets, implying that at small length scales classical PR theory is not wholly sufficient to predict interface stability^16^. This too is the case in liquid nanowires (see supplementary details).

**Figure 5:**
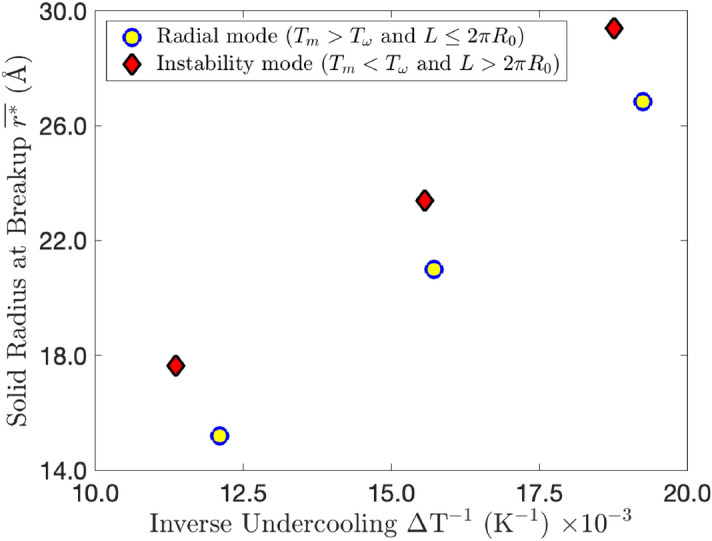
Values of $r^*$ obtained from simulation against $\Delta T^{-1}$. Yellow circles are for $r^*$ when $L \le 2\pi R_0$, and red diamond points represent $r^*$ and $\Delta T$ for when $L > 2\pi R_0$. Yellow circles indicate when the radial mode is the preferred melting mechanism, and red diamonds indicate where the instability mode is preferred. The first two points (bottom left) are for wires where $R_0 \approx 22$ Å  the middle two are for $R_0 \approx 30$ Å, and the last two are for $R_0 \approx 38$ Å. We assume yellow and red points represent the bulk and instability melting temperatures, $T_{\text {m}}$ and $T_{\omega }$, respectively.

We now examine how the wire length influences the stability of the solid-liquid interface by looking at how $r^*$ obtained from simulation behaves close to the melting point for wires of different radii and lengths. From Eq. (2) we can see that $r^* \propto \Delta T^{-1}$. As observed in Fig. 5, MD results consistently show that longer wires melt at a lower temperature since they are prone to the growth of instabilities that initiate the pinch-off. Shorter wires not only melt at a slightly higher temperature, but the value of $r^*$ at the point when the pinch-off initiates is consistently smaller, in agreement with the theory developed. It supports the idea that there are two melting mechanisms that depend on the wire aspect ratio, as evident in Fig. 4. The simulated results point to the bulk melting temperature of the potential used being between 1220 and 1230 K, rather than the 1320 K stated.

**Figure 6:**
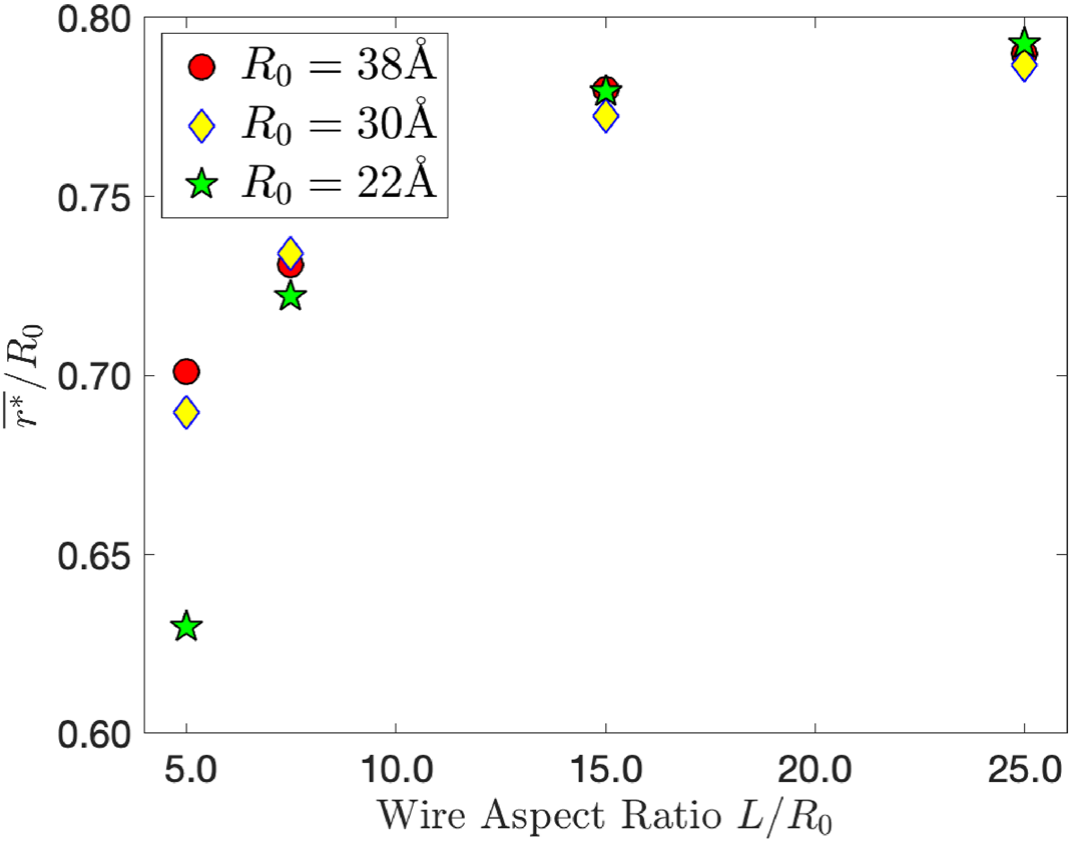
The ratio of equilibrium solid radius to initial wire radius against the wire aspect ratio. Values of $r^*/R_0$ for each aspect ratio are clustered closely together, where they begin to deviate markedly when the initial wire length $L < 2 \pi R_0$.

In Fig. 6 we see that $r^*$ depends not only on the initial wire radii $R_0$, but also on the wire aspect ratio as well. This evidence supports the theory and previous claims that as the wire aspect ratio gets smaller, the solid core radius prior to the pinch-off decreases. The ratio of $r^*/R_0$ appears to converge in the limit of large $L/R_0$. Each $r^*/R_0$ for the aspect ratios studied are similar when $L> 2\pi R_0$. Once $L \le 2\pi R_0$ the difference in $r^*/R_0$ becomes appreciable. This could be indicative that quantities like the interfacial energies and correlation length ($\gamma _{\text {sl}}$ and $\xi$ respectively) become important quantities for small wires, implying size and curvature play key roles in observations for small aspect ratios. (See supplementary details for the mean and standard deviations for $T_{\text {m}}$, $r^*$ and $k_{sol}r^*$).

## Discussion

By perturbing the interface of a surface melted metal nanowire, we can describe the existence of two mechanisms for nanowire melting; an instability mode or radial mode. The model showed the fastest growing modes that destabilise the interface are inversely proportional to the wire length, where a PR type instability for the solid in a surface melted nanowire is recovered. By using classical nucleation theory and exploring the nanowire stability in the vicinity of $T_{\text {m}}$, we were able to define the condition that determines the preferred melting mechanism. Moreover, we recovered an expression for the equilibrium solid radius in terms of the initial wire radius and the interplay of the interfacial energies of the nanowire.

Simulations show the fastest growing modes are inversely proportional to the wire length, and in fact that $k_{sol}r^* \propto \frac{2\pi R_0}{L}$. Additionally, we observe longer nanowires consistently melt at a lower temperature than shorter wires, in agreement with our developed theory and other recent observations^23^. The implication is that shorter nanowires have a more stable interface when close to their melting temperature. For nanowires where the instability mechanism is the preferred melting mode, once the pinch-off has initiated the remaining solid will be consumed. This is because the solid core tries to stabilise itself by forming into a sphere, minimising its surface energy. In some cases it was observed that for the longest, thinnest nanowires, the liquid-vapour interface would begin to neck, being driven by surface diffusion, which in turn influenced the breakup of the solid. For longer heating rates or overdamped Brownian dynamics, this feature would become more pronounced. However, due to the quick equilibration at each timestep, this was not an issue.

Evidence can also be seen that $r^*$ depends on the wire aspect ratio and not just the initial radius. This has been reported in previous work, where for nickel and aluminium nanowires of a single length but increasing radii, the solid core remained stable down to smaller radii^8^. Studies have explored the size-dependence of interfacial energies^35^. Our study, however, shows surface area of metal nanowires becomes an important factor in interfacial energies for small wire lengths. Curvature too plays a role in the interfacial energy, where for spherical clusters the solid-liquid interface energy is linear with inverse radius^36,37^. This size and curvature dependence explains why values of $r^*/R_0$ begin to deviate away at low aspect ratios. The ratio of atoms at the surface compared to the bulk becomes far more appreciable for the smallest wires, giving the curvature a greater role in the solid-liquid interface dynamics. Given the fact that the fastest growing modes are inversely proportional to the nanowire length, it would be of no surprise that interfacial energies will depend on this too since their surface area will scale with radius and length. The theory and simulations show that long nanowires are thermodynamically unstable at high temperatures since the nanowire length will almost always be much greater than its equilibrium solid radius. This has ramifications when considering device stability that utilise nanowires subjected to heating. We observed that for long, thin nanowires, the liquid-vapour interface can begin to destabilise even before the solid begins to neck. This implies ultra-long, thin nanowires will be particularly unstable at elevated temperatures and should be considered when constructing nanowire devices.

## Conclusion

We studied the stability of the solid in copper nanowires as they approach their melting temperature by perturbing a model describing interface kinetics and compared the results to MD simulations. The model found a stability criterion that dictates the preferred melting mode a nanowire will take. We found that longer nanowires are thermodynamically unstable, and will preferentially pinch-off and melt, indicating a melting mechanism driven by a PR type of instability. In shorter nanowires, the interface front moved radially towards the nanowire centre before the solid would breakup, indicating higher interface stability, with MD results in agreement with our model. Moreover, we proposed modes that destabilise the solid-liquid interface are dominated by the nanowire length, in contrast to PR theory which states they are proportional to the circumference. Additionally, it was observed from the MD simulations that longer nanowires consistently have a melting temperature a few degrees below shorter nanowires, indicating the nanowire aspect ratio influences the preferred melting mode and solid-liquid interfacial stability.

## Supplementary Information

Supplementary Information.

## Data Availability

The datasets used and/or analysed during the current study are available from the corresponding author on reasonable request.
