# Supplementary material for: Nanowire melting modes during the solid–liquid phase transition: theory and molecular dynamics simulations
Source: Sci Rep. 2022 Nov 21;12:20052. doi: 10.1038/s41598-022-24654-z (PMC9681868; doi:10.1038/s41598-022-24654-z)
Supplement: Supplementary file 1 — Supplementary Information. [file 41598_2022_24654_MOESM1_ESM.pdf]

Supplementary Materials for Nanowire melting modes during the Solid-Liquid Phase Transition: Theory and Molecular Dynamics Simulations

KANNAN M. RIDINGS[1,*] AND SHAUN C. HENDY[2]

[1] *The MacDiarmid Institute for Advanced Materials and Nanotechnology, Department of Physics, The University of Auckland, Auckland 1010, New Zealand*
[2] *Toha Foundry, Auckland, 1025, New Zealand*
[*] *k.ridings@auckland.ac.nz*

1. S1 INTRODUCTION

Supplementary information for the paper *Nanowire melting modes during the Solid-Liquid Phase Transition: Theory and Molecular Dynamics Simulations* is contained in this document. It includes details of the molecular dynamics (MD) simulations and the computational methodology used in the analysis of the MD simulations presented in the accompanying paper. Additionally, it features the same analysis used on the solid-liquid interface to the liquid interface. All atomistic visualisations were made with Ovito [1].

2. S2 COMPUTATIONAL DETAILS

FCC nanowires are bounded by {100} and {110} surfaces, which are made via a Wulff-type construction (see [2] Fig. 2). This ensured that the initial state of each nanowire is bounded by the same surfaces, and have approximately the same proportion of atoms on the {100} surfaces to the {110} surfaces. This helps to rule out any differences in observations due to the presence of more (or less) atoms on one surface compared to another, which can lead to differences in surface energies.

To account for the expansion of the lattice, an atomic volume of approximately $V_{\text{atom}} = 13.2\text{Å}$ was used. This assumed the density of copper at the melting point was $\rho_{Cu} = 8020\ \text{kg/m}^3$.

In this study, a Langevin thermostat was used to control the temperature. The features of this that make it favourable is that it introduces a random force to the system that is proportional to the temperature. Moreover, there are no correlations with former velocities or forces [3]. This means that there is no net force that accumulates that could generate bad dynamics of the sample.

The phase of each atom was determined by modified Steinhardt parameters [4, 5]. Looking at Supplementary Fig. S1 we see a bimodal distribution of $\overline{q_6}$, with one peak being found at $\overline{q_6} \approx 0.189$, and another at $\overline{q_6} \approx 0.417$. This helps define a threshold of $\overline{q_{cut}} \approx 0.300$, where if $\overline{q_6} > \overline{q_{cut}}$ the atom is more likely to be in a solid phase, and if $\overline{q_6} < \overline{q_{cut}}$ the atom is more likely to be in a liquid phase. We then use temporal averaging to reassign potential 'misclassified' atoms. We look at atoms at a time t_i and t_{i+1}, and if atoms have switched phases from time t_i to time t_{i+1}, we reassign the atom the phase it had at time t_i. This clears up most misclassified atoms in the system.

To estimate values of the melting temperature, we calculated heat capacity $C_{\text{v}} = \frac{dE}{dT}$ and use the peak to identify the bulk melting temperature of each wire, where the C_{v} diverges $T \to T_{\text{m}}$. These were averaged across multiple runs for each wire to give a reliable estimate of the melting temperature for each wire.

3. S3 SUPPLEMENTARY MD DETAILS

This section contains additional information on the analysis of the MD simulations used to construct the arguments in the main manuscript. We first look at a table of the main quantities calculated in this study. Supplementary Table S1, we see the mean and standard deviation for

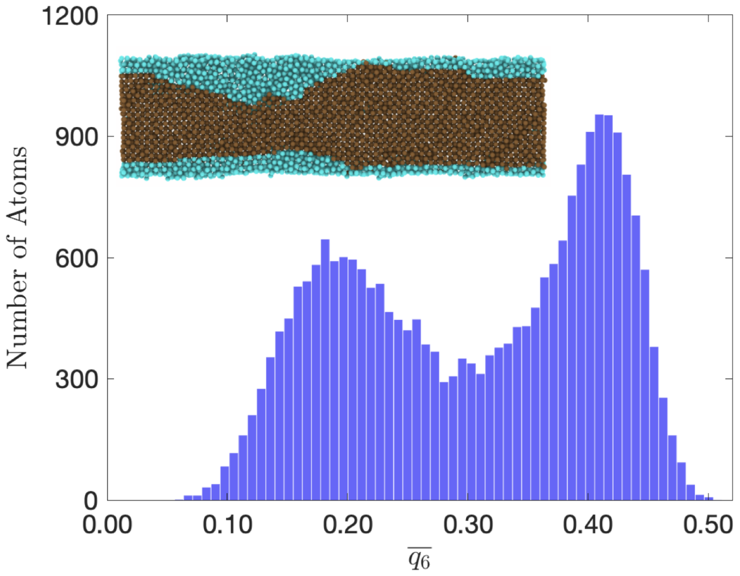

Supplementary Figure S1. This figure shows the bimodal distribution of the $\overline{q_6}$ order parameter during the solid-liquid phase transition with an accompanying snapshot.

the quantities of interest in this study for each wire radii and length studied: the bulk melting temperature of each wire; the radius of the solid right before the breakup; the fastest growing modes that destabilise the solid-liquid interface. From Fig. 5 in the main text, and the data presented in Supplementary Table S1, we see that shorter wires tend to have a slightly higher melting point than longer wires due to the stabilisation of the interface. This is particularly noticeable for the thinnest nanowires with $R_0 \approx 22$ Å, but less so for the $R_0 \approx 30, 38$ Å. For values of r^* we see that their values are the lowest for the shortest wires, indicating that more solid is required to melt radially before the solid breakup can take place. For the longest wires, we see that the values for r^* are similar, indicating that right before the solid breakup, the interface can not promote more nucleation at its surface, and so it must melt via the growth of an instability that breaks the solid apart. For the fastest growing modes we see a general trend that follows the scaling relation $k_{sol}r^* \propto \frac{2\pi R_0}{L}$. This shows that the fastest growing modes that destabilise a solid-liquid interface do not strictly follow a Plateau-Rayleigh instability, where the preferred mode depends only on the wire circumference and not its length. To study the interface stability, we averaged binned values of $r^*(z)$ along the wire axis prior to the solid pinch-off to obtain an estimate of $\bar{r^*}$. A nearest-neighbour algorithm is then used to extract atoms at the solid-liquid interface. Once all the atoms in the solid are appropriately classified, we can check how many neighbours each atom has within a range r_{cut}, where r_{cut} is generally chosen to be within the first or second nearest-neighbour shell. This can isolate most atoms at the interface to estimate the solid radius, r^*, at a point along the wire axis. The atoms at the solid-liquid interface are then binned along the axis of each wire so that an estimate of $r^*(z)$ can be obtained. Once $r^*(z)$ is calculated, we can compute the Fourier transform of the interface profile. When calculating the Fourier transform of a given interface, a sampling frequency $f_s = 4$ was chosen since this was the nearest whole number to the lattice spacing of copper that was used in this study. In Supplementary Fig. S2), the distance between the top layer of the interface profile and the bottom layer is approximately 4, justifying our choice of sampling frequency.

The Fourier transform of $r^*(z)$ was taken to extract modes that destabilised the solid-liquid interface. Destabilising modes of k_{sol} (k_{max} for the solid-liquid interface) were averaged across all runs, giving a more reliable estimate of the Fourier profile for each wire. Supplementary Fig. S3 shows the averaged Fourier transform for all wires of initial radii $R_0 = 38$, 30, 22 Å, with each satisfying $L/R_0 = 25$. This is obtained by taking the Fourier transform of the solid profile, seen in Fig. 3 of the main text. The Fourier transform presented in Supplementary Fig. S3 represents averaged the Fourier transform across all runs for the wire lengths mentioned above. The dashed lines indicate where a data point for $k_{sol}r^*$ are extracted from, given the Fourier transform from

$R_0 \approx 22$ Å	T_m (K)	r^* (Å)	$k_{sol}r^*$
L_1	1144.7 ± 0.6952	14.2 ± 1.05	0.487 ± 0.0638
L_2	1140.1 ± 1.866	16.2 ± 0.548	0.349 ± 0.0452
L_3	1137.8 ± 1.868	17.5 ± 0.335	0.205 ± 0.0889
L_4	1136.2 ± 1.684	17.8 ± 0.267	0.282 ± 0.144

$R_0 \approx 30$ Å	T_m (K)	r^* (Å)	$k_{sol}r^*$
L_1	1161.4 ± 2.570	20.0 ± 1.74	0.532 ± 0.0760
L_2	1160.9 ± 1.718	22.0 ± 0.645	0.395 ± 0.0447
L_3	1160.6 ± 0.9416	23.2 ± 0.337	0.199 ± 0.0630
L_4	1161.2 ± 0.9675	23.6 ± 0.246	0.272 ± 0.125

$R_0 \approx 38$ Å	T_m (K)	r^* (Å)	$k_{sol}r^*$
L_1	1172.7 ± 1.545	26.3 ± 1.68	0.585 ± 0.0696
L_2	1173.4 ± 1.163	27.4 ± 1.03	0.359 ± 0.0549
L_3	1171.4 ± 0.7098	29.2 ± 0.448	0.255 ± 0.0951
L_4	1172.0 ± 0.5714	29.6 ± 0.456	0.158 ± 0.0584

Supplementary Table S1. Three tables for the initial wire radii $R_0 \approx 22, 30, 38$ Å respectively. For each wire length investigated, we included the mean and standard deviations for the bulk melting temperature of the wires T_m, the radius of the solid right before the breakup r^*, the fastest growing mode which destabilse the solid-liquid interface $k_{sol}r^*$.

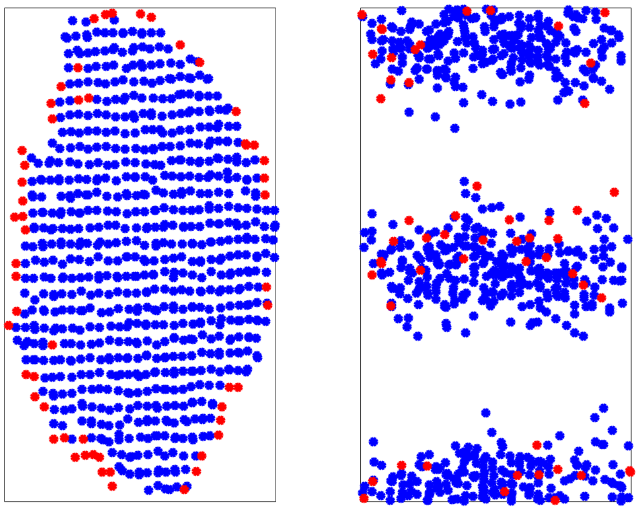

Supplementary Figure S2. This is an example of how the solid radius, r^* is defined. On the left is a slice of the xy-plane for one sampling of the interface, and on the right is the view down the xz-plane. The red points are used to approximate $r^*(z)$ by averaging over all the points in that segment of the wire.

a single MD run. These data points for $k_{sol}r^*$ are averaged across all MD runs for a given wire aspect ratio, as can be seen in Fig. 4 of the main text.

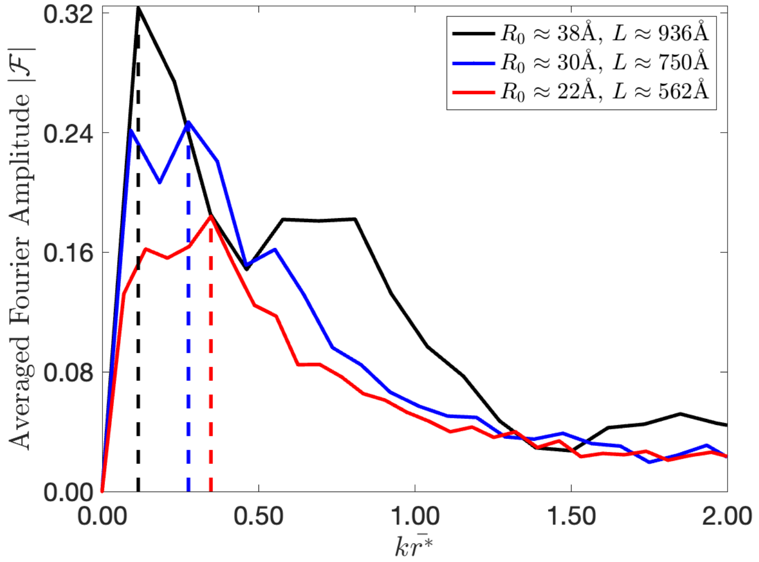

Supplementary Figure S3. The averaged Fourier transform of wires with $L/R_0 = 25$. Values of k_{sol} for each wire are all low, indicating modes that destabilise the solid-liquid interface are proportional to the wire length. Dashed lines indicate the points where $k_{sol}r^*$

Supplementary Fig. S4 shows the interface profile of a liquid nanowire of length $L = 1084$ Å as it begins to break up. R_0 represents the initial wire radius, and $R(z)$ is the instantaneous radius of the liquid nanowire along its length z. The interface profile is smooth due to the presence of higher surface tension (when compared to the solid-liquid interface). The necking of the wire is caused by the atoms in the neck migrating away to increase their coordination number.
In Supplementary Fig. S5 we see the Fourier transform for a wire of radius $R_0 \approx 30$ Å and length $L = 1084$ Å, which is calculated by taking the average of 25 individual MD runs. This shows a distinct mode destabilising the liquid interface. This can particularly be seen when we compare it to Supplementary Fig. S3, where modes that destabilise the solid-liquid interface appear to be proportional to the wire length.
Next, we see Supplementary Fig. S6, the stability diagram for the liquid in terms of the fastest growing modes $k_{liq}R_0$ (where k_{liq} is k_{max} for the liquid) against the wire aspect ratio L/R_0. In the case of the liquid, wires shorter than a critical length (where $L_{crit} \simeq 2\pi R_0$), the breakup of the liquid interface will not happen.
PR theory predicts that $k_{max}R_0 \approx 0.697$ (or $\lambda \approx 9.01R_0$). Supplementary Fig. S6 suggests that at the nanoscale, this number is lower, where it is found that $k_{liq}R_0 \approx 0.423$ (giving $lambda \approx 14.85R_0$). This is slightly higher than what has been reported previously [6], but consistent with the result that PR theory over predicts the fastest growing modes at the nanoscale [7].

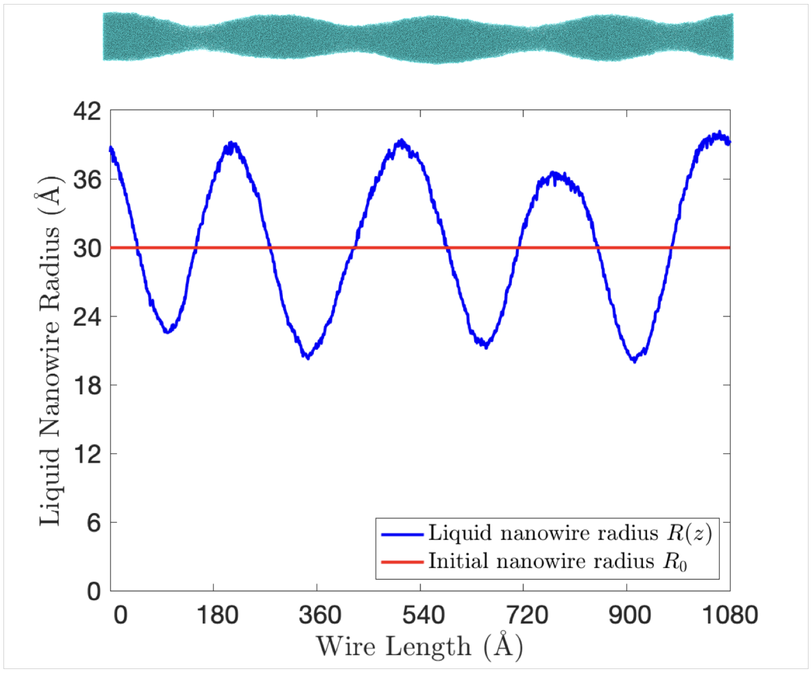

Supplementary Figure S4. A single MD run showing how the liquid nanowire radius $R(z)$ varies across its length, with an accompanying snapshot. Marked in red is the initial wire radius R_0. The interface profile here is smooth, with the undulations in the wire clearly represented when compared to the snapshot.

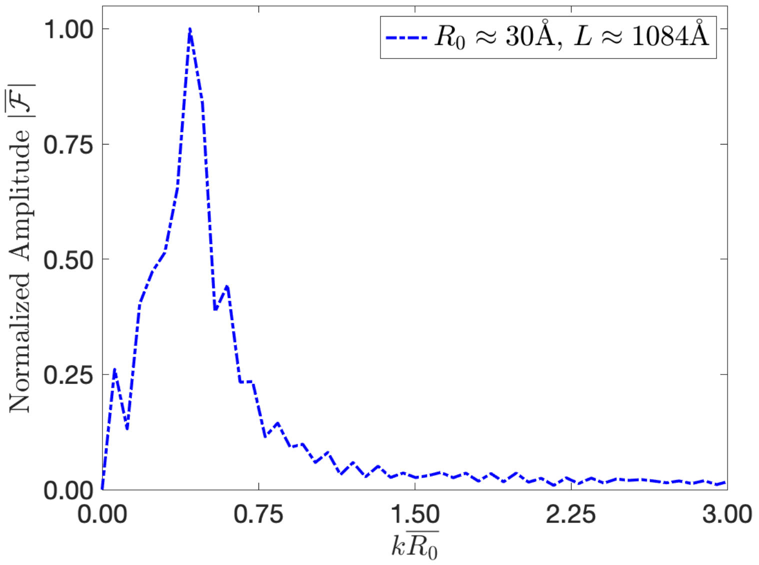

Supplementary Figure S5. The Fourier transform of the liquid interface profile has been calculated for 25 individual MD runs, and is averaged across all simulations. A clear distinct mode can be seen giving a value of $k_{liq}R_0 \approx 0.421$.

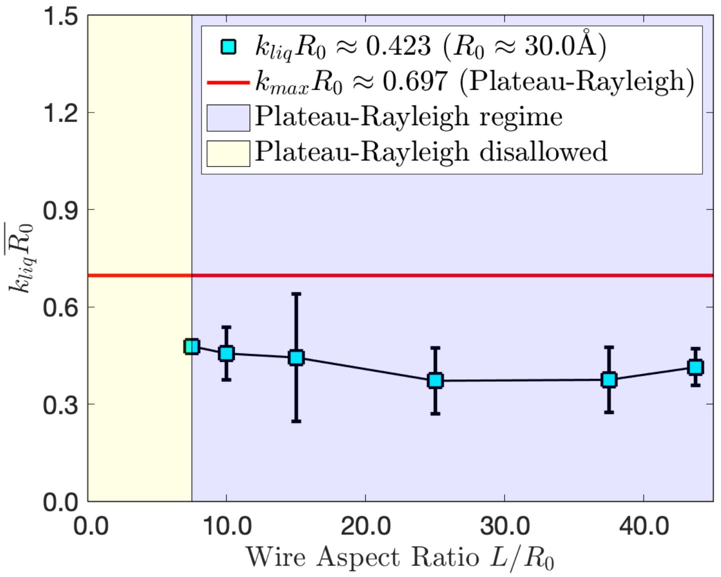

Supplementary Figure S6. This figure shows how modes that destabilise the liquid interface change with the wire aspect ratio. As we can see, the relationship is approximately flat, showing that $k_{liq}R_0 \approx 0.423$ for a liquid nanowire. The blue shaded region shows where liquid wires are unstable to perturbations, and the yellow shaded region shows where the PR breakup of the interface is disallowed.

REFERENCES

1. A. Stukowski, "Visualization and analysis of atomistic simulation data with ovito–the open visualization tool," Model. simulation materials science engineering **18**, 015012 (2009).
2. K. M. Ridings, T. S. Aldershof, and S. C. Hendy, "Surface melting and breakup of metal nanowires: Theory and molecular dynamics simulation," The J. chemical physics **150**, 094705 (2019).
3. W. Van Gunsteren and H. Berendsen, "Algorithms for brownian dynamics," Mol. Phys. **45**, 637–647 (1982).
4. P. J. Steinhardt, D. R. Nelson, and M. Ronchetti, "Bond-orientational order in liquids and glasses," Phys. Rev. B **28**, 784 (1983).
5. W. Lechner and C. Dellago, "Accurate determination of crystal structures based on averaged local bond order parameters," The J. chemical physics **129**, 114707 (2008).
6. J. Eggers and T. F. Dupont, "Drop formation in a one-dimensional approximation of the navier–stokes equation," J. fluid mechanics **262**, 205–221 (1994).
7. C. Zhao, J. E. Sprittles, and D. A. Lockerby, "Revisiting the rayleigh–plateau instability for the nanoscale," J. Fluid Mech. **861** (2019).
